# Advantages of doubly polished thin sections for the study of microfossils in volcanic rock

**DOI:** 10.1186/1467-4866-7-5

**Published:** 2006-05-30

**Authors:** M Ivarsson

**Affiliations:** 1 Dept. of Geology and Geochemistry, Stockholm University, Stockholm, Sweden

## Abstract

Doubly polished thin sections, originally prepared for fluid inclusion studies, present great advantages in the study of microfossils in volcanic rocks. Better visibility and light conditions, variation in thickness of the thin sections and the possibility to combine fluid inclusion studies with microfossil studies lead to a wide range of advantages over ordinary thin sections. This includes the study of morphology, internal microstructures, colonies, association with the substrate that microfossils are attached to and geological and environmental context in which the microfossil once lived. When meeting the criteria of microfossil recognition the advantages of doubly polished thin sections are substantial and can be crucial in distinguishing between biogenic microfossils and abiotically formed abiomorphs.

## Introduction

Knowledge of the deep subsurface biosphere is constantly increasing, which creates a need for new analytical methods. Only during the last decade has it been evident that lithoautotrophs exist deep in the ocean crust in association with hydrothermal systems [1,2]. Such bacteria are found attached to volcanic glass or mineral surfaces using elements like Fe or Mn for their metabolism.

When studying fossilized microorganisms in hard rock, optical microscopy of thin sections is an essential tool because it is the only technique to study the isolated interior of rock samples without contamination and it also facilitates morphological and microstructural studies [3]. In this paper the use of doubly polished thin sections (sections that are polished on both sides) for studies of microfossils is emphasized. Doubly polished thin sections have somewhat different properties that make them more suitable for the study of microfossils compared to ordinary thin sections. First of all, it is possible to vary the thicknesses of the thin sections to fit the size of the observed feature. Usually they are between 150 and 200 μm compared to ordinary thin sections that normally are ~30 μm. Furthermore, they can be viewed under the microscope without being mounted on a microscopic glass slide. Thus, they offer better light conditions, higher visibility and an increased three-dimensional view.

In this study rock samples collected during the Ocean Drilling Program (ODP) Leg 197 at the Emperor Seamounts in the Pacific Ocean have been used. During that Leg three different seamounts were drilled, Detroit, Nintoku and Koko Seamount, respectively, and four sites were drilled: Site 1203 (50° 57.00'N, 167° 44.40'E) and Site 1204 (51° 11.68'N, 167° 46.36'E) at Detroit Seamount, Site 1205 (41° 20.00'N, 170° 22.70'E) at Nintoku Seamount and Site 1206 (34° 55.55'N, 172° 8,75'E) at Koko Seamount. Basalt samples from all four sites were used in this study. Emperor Seamounts is a hotspot track that results in increasing ages away from the active volcanic center which is today the Hawaiian Islands. The most recent offspring of this volcanic chain is Loihi Seamount south of the Hawaiian Islands. The ages of the sampled seamounts is ~81 Ma for Detroit Seamount, ~56 Ma for Nintoku Seamount and ~48 Ma for Koko Seamount [4].

## Conditions in volcanic rock

The greatest difference between sedimentary rocks and crystalline rocks, in the study of microfossils, is that in sedimentary rocks microorganisms may occur throughout the samples, but in crystalline rocks the occurrence of microorganisms is limited to veins and the most adjacent parts of the host rock. They exist in open fractures as long as hydrothermal fluids are circulating. As soon as the veins are completely filled with minerals the microorganisms die and may be fossilized. Usually the microorganisms are found either attached to the vein walls or within the substrate in biodisolved cavities [1]. It has also been shown that microorganisms can be attached to secondary minerals like zeolites [5,6]. When microfossils are found attached to a surface they are usually preserved in secondary vein-filling minerals like calcite. This is a problem when performing elemental analysis for carbon and other elements that can indicate a biogenic origin, for example P or N, may be of importance instead.

A variety of morphological types have been reported from volcanic rocks in ocean crust: coccoids [6], branched filaments [7], hollow sheaths [8] and twisted stalks [9]. Most have been interpreted as iron-oxidizing bacteria. Compared to photoautotrophs, that use sunlight as their source of energy, lithoautotrophs use chemically stored energy in minerals as energy source. In volcanic rocks iron is the most abundant and prominent element as an energy source for their metabolism, but elements like, for example, Mn also appear to be involved in the metabolic process.

Determination of whether putative microfossils truly represent past life, or if they consist of abiotic fossil-like structures is problematic. The term biomorph was coined by Garcia-Ruiz and co-workers [10] for abiotically formed features similar in morphology and composition to putative microfossils found in ~3.5 Ga Apex Chert in Warrawoona Group of northwestern Australia, originally regarded as the oldest microfossils found on Earth [11]. Garcia-Ruiz and co-workers succeeded in producing carbonate-silica aggregates with kerogenous coatings and claimed that such biomorphs could offer an alternative explanation for the genesis of the Apex microfossils. Hofmann [12] subsequently pointed out that the correct term for an abiotic entity that mimics biological morphology should be abiomorph, not biomorph.

Biogenicity of Archean microfossils has been discussed for several decades, ever since they were first found, and the Apex chert microfossils are a good example of such a controversy. They have been interpreted very differently by Schopf [11] and Schopf et al. [13] compared to Brasier et al. [14,15]. Schopf [11,13] claimed that the putative microfossils represented preserved filamentous cyanobacterium-like microorganisms found in a bedded chert unit of the Early Archean Apex Basalt. He described carefully and in detail the morphology of the microfossils and interpreted that they represented eleven different biological taxa. Brasier and co-workers [14,15], on the other hand, claimed that the purported microfossils were secondary artifacts formed from amorphous graphite. They also re-interpreted the geological context, suggesting that the Apex chert was a hydrothermal setting. The occurrence of isotopically light carbon compounds were explained as a result of Fischer-Tropsch-type synthesis, which is also consistant with the hydrothermal model.

The biogenicity of microfossils and traces of microfossils found in volcanic rocks of modern ocean floors is less controversial than that of Archean microfossils. The reason is perhaps because they are a relatively new phenomenon and not really established in the scientific community yet, but probably more due to the extreme scientific and public interest of early Archean microfossils. Acceptance of such extraordinary findings appears to require extraordinary evidence. However, the discovery of biomarkers in pillow lavas from the ~3.5 Ga old Barberton Greenstone Belt (BGB), South Africa [16,17] may change this. These findings indicate that subaqueous volcanic basalts provided niches for early life on Earth, perhaps the earliest. Also, observations of probable bioweathered tunnels in olivine and pyroxenes from the Martian meteorite Nakhla that are similar in appearance to microstructures in terrestrial basalts associated with microbial activity may further stimulate this debate [18].

### Criteria for biogenicity

To verify that a microfossil is biogenic in origin and not abiogenic, some criteria have been established and used by the scientific community [3,19,20]. Usually such criteria are formulated to fit Archean samples with a sedimentary origin, but the following is a summary of such criteria adjusted to suit samples from volcanic rocks of all ages based on experiences made from working with doubly polished thin sections:

1. Geologic context of the sample must be known; is it compatible with past life? Age of the rock is of interest to relate possible life to geologic history but not crucial since the microfossils are not syngenetic with the rock.

2. Is the putative microfossil indigenous to the rock? (rather than being a modern contaminant). Is the indigenous microfossil syngenetic with secondary minerals? Did it exist while the hydrothermal system was active?

3. Does the sample contain evidence of microbiological morphology?

4. Is the fossil-like microstructure biogenic? Is there any evidence of stable isotope patterns unique to biological systems? Are any organic biomarkers present?

5. Is there evidence for structural remains of colonies or communities within the sample?

6. Is there any evidence of biominerals?

Among these criteria number four, establishment of biogenicity, is the most difficult to fulfill. Biogenicity of a putative microfossil can rarely be determined by morphology alone. It is preferable to combine morphological studies with chemical analysis to determine authenticity of a microfossil. Buick [3] discussed at what level of morphological complexity an object can be considered to be an undoubted microbial relic. Since simple coccoid-like spheroids and filament-like structures can be produced inorganically by weathering, abiotic precipitation or displacive growth of diagenetic crystal chains, Buick [3] emphasized that the objects must show further structural elaboration indicative of cellular morphology. Spheroids should show layered walls, sculptured surfaces, internal kerogen bodies or regular group organization. Partitioned tubes should have septae subdividing them into regular cylindroids, and hollow tubes should have layered walls, trichome remnants or longitudinal variations in width and shape before being considered biogenic.

Considering abiomorphs [10], morphology alone can never be sufficient to determine biogenicity. However, the physicochemical conditions required for the self-assembly of such abiomorphs are rare in nature. The reaction requires ambient temperature, an alkaline medium (up to pH 11), silica and carbonate sources, barium ions as well as organic precursors. Even if this reaction is possible in nature it must be very rare. Environments like hydrothermal systems associated with serpentinization of ultra mafic rocks could perhaps meet up to such criteria [21,22]. However, the need of ambient temperature for the reaction to proceed will not be fulfilled since hydrothermal systems by definition are characterized by elevated temperatures. This means that the first criterion regarding geological context is very important. Knowledge about the geological and geochemical conditions can tell whether the conditions are preferable for abiomorph formation or not. Similar abiomorphs could possibly form under different conditions out of different elements but so far no experiments can confirm that. This means that it is probable that a putative microfossil with well preserved structural elaboration should be biogenic rather than abiogenic. The importance of knowledge about the geological context is also obvious considering the controversy between Schopf [11,13] and Brasier et al. [14,15].

**Figure 1:**
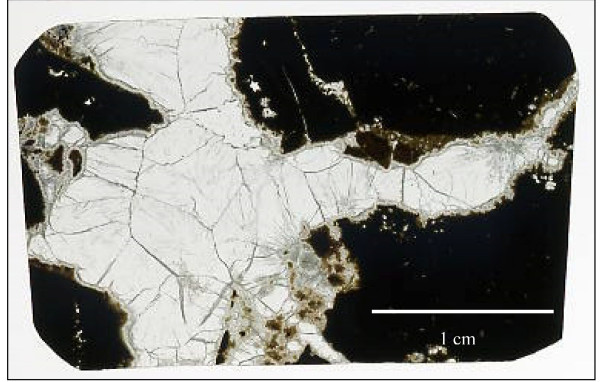
A doubly polished thin section showing calcite filled veins in volcanic rock.

The best method to study fossilized microorganisms associated with veins is by using thin sections (Fig. 1). They represent an unaffected cross-section of the sample that has not been exposed to any surrounding environment since the filling of the vein by secondary minerals, usually some carbonate mineral such as calcite or aragonite. All microfossils found attached to the vein walls and embedded within secondary minerals or the host rock are syngenetic with the minerals. Buick [3] actually had as his first criterion that a putative object should occur in a petrographic thin section.

## Doubly polished thin sections

Usually, an ordinary thin section is ~30 μm thick and mounted on a transparent glass microscope slide. Since microfossils can range over 30 μm in size, this thickness is obviously quite limiting. Doubly polished thin sections are polished on both sides to provide the best light conditions possible. The thickness of doubly polished thin sections (Fig. 1), may be varied to fit the requirements of the study. Commonly they are ~150–200 μm thick and are, therefore, more suitable when detecting and studying microfossils. Below 150 μm thin sections tend to get too fragile and crack easily if they are not mounted on a glass slide. Doubly polished thin sections were originally used in the study of fluid inclusions, which commonly occur in approximately the same size range as microfossils. Hence, the ~150–200 μm thickness is optimal for the study of structures and textures in the size range of interest [23] making doubly polished thin sections very suitable for both purposes.

The ideal thickness of the thin sections is dependent on a wide range of factors. The grade of anisotropy is crucial. Microstructures will be much easier to resolve in a weakly anisotropic mineral than in a strongly anisotropic mineral. Grain size of the minerals is another influential factor, since small grain size will reduce the light transmission. Also, the density of inclusions of the host mineral will affect the visibility. A high density of inclusion will impede the light transmission. The thicker the thin section the better is the chance to detect microfossils, but increased thickness will also reduce visibility into the thin section interior. In the current study it was found that ~200 μm represents a good upper limit. Above that thickness loses in visibility and light conditions make it difficult to distinguish small textures in detail. A 200 μm thickness is also enough to detect several complete microfossils in the same thin section. It is enough to study a colony of fossilized bacteria. Another advantage with thicker-than-normal thin sections is the three-dimensional view obtained. This facilitates the study of microfossil morphology and the association with mineral surfaces. It eases the study of vein textures and mineral properties that may provide valuable information for living conditions as well as metabolic processes. This is also a question which needs careful consideration since an increase in thickness leads to an increase in a three-dimensional view. It is, thus, crucial to find a balance between thickness, light conditions, visibility and a three dimensional view that is suitable for the analysis.

It should also be noted that thickness of the sections is not responsible for the visibility and quality of the image. Doubly polished thin sections are much more difficult to prepare than ordinary thin sections, and therefore the polish preparation is crucial for the quality of the image. Any imperfections on the surface serve to disperse the light and degrade the quality of the image. Remnants of glue on the surface or incorporations of glue in the sections from the preparation also result in reduction of visibility and quality.

**Figure 2:**
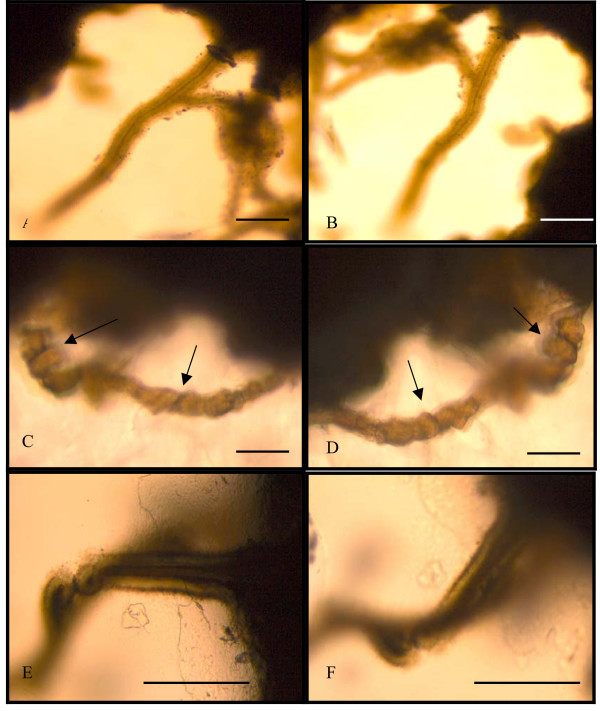
Photos showing microfossils from opposite sides. An example of the advantages of using both sides of a thin section. A and B showing a tubular filament attached to volcanic glass from opposite sides. C and D showing a twisted filament where the same twisted features are shown from both sides indicating that it is a three-dimensional morphological feature. E and F showing a filament blocked by another filament. In E the whole filament is visible but in F parts of the filament is blocked. The scale bars are 10 μm.

**Figure 3:**
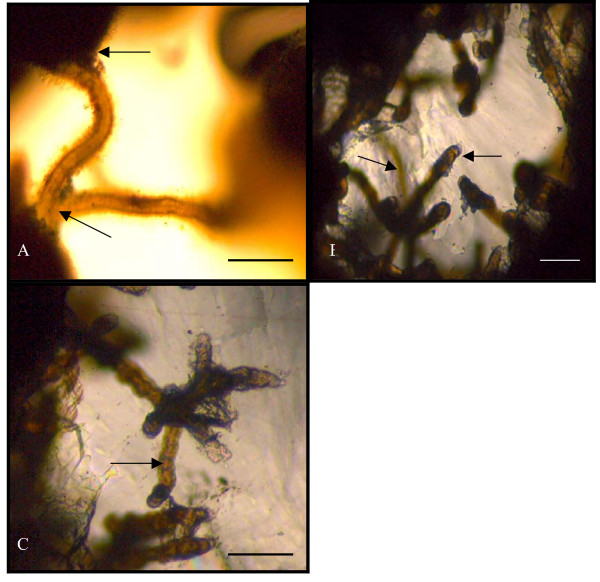
A showing the way of attachment of the microfossil to the substrate. Arrows showing how the filaments are attached at both ends to a surface. B showing a colony of fossilized bacteria filaments. Arrows showing filaments at different levels of sight within the thin section. C showing internal morphology of a tubular filament. Scale bars 10 μm.

**Figure 4:**
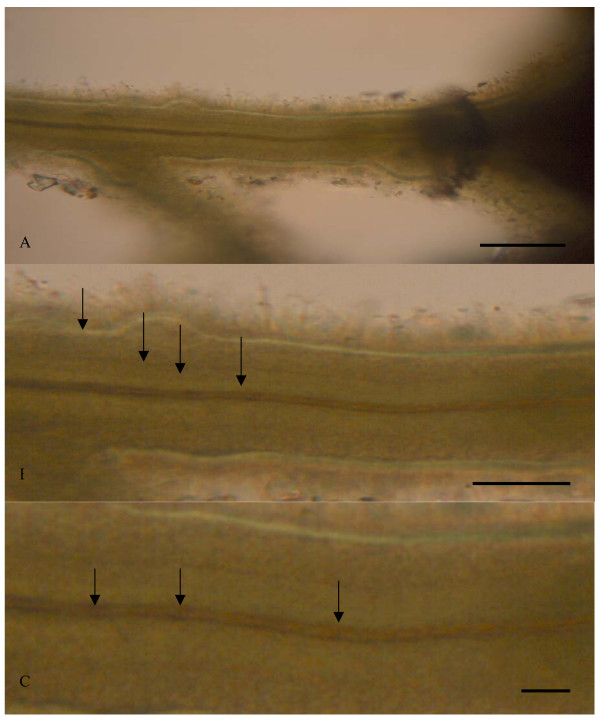
Microphotograph of close-up pictures of the filament in figure 2A and B showing internal microstructures. Note the different layers and the variations of each layer. Arrows in figure B marking three parallel layers within the filament and a tube in the middle. Arrows in figure C marking possible trichome remnants in the tube, small spheroidal features. Scale bars in A: 10 μm, in B: 5 μm and in C: 1 μm.

However, the second advantage of using doubly polished thin sections is that they are indeed doubly polished. This means that they are not mounted on a transparent slide and can therefore be examined from both sides. This in turn makes it possible to view microfossils from both sides and to obtain a three-dimensional image of the fossil (Fig. 2A–F). Morphology and microstructures of microfossils like twisted stalks or hollow sheaths become much easier to study (Fig. 2, 3, 4), and if morphological features are diffuse on one side they may be more prominent on the other side (Fig. 2C–F). Doubly polished thin sections actually provide an opportunity to confirm that a morphological feature of a microfossil is a three-dimensional feature that appears on both sides and not only on one side (Fig. 2A–F). They also give the rare opportunity to study common features or differences within accumulations of microfossils. This includes surface structures and morphology, way of attachment to a surface (Fig. 3A) and differences in mineral surfaces, differences in species within a colony as well as internal relationships between different individuals. In an accumulation of microfossils, some individuals can get blocked by others when viewed from one side but may be seen from the other side (Fig. 2E, F). Due to the greater thickness of the doubly polished thin sections bacteria filaments in a colony occur at different focal depths within the thin section (3B).

Figure 4 illustrates an example of the advantages of using doubly polished thin sections in the study of internal morphology and microstructures of microfossils. This figure shows close-ups in various degrees of the bacteria filament from figures 2A and 2B. In these close-up pictures it is possible to observe at least three internal zones, with various appearances, separated by layered walls (4B). The outermost zone is more heterogenous than the second one that is quite homogeneous in appearance. The innermost zone represents a tube-like structure. This tube is most possibly hollow, and when studying it in detail it is possible to observe spheroidal features that most likely represent trichome remnants.

The filament, including the two outermost zones, is quite thick compared to the innermost tube. Normally, the tube occupies most of the space of a bacterial filament, and the walls represent a minor part of the filament. The thick walls of this filament may be explained by mineral deposition within as well as onto the filament. Several iron-oxidizing bacteria are known to deposit oxidized iron within their sheaths and stalks [24].

The third advantage of using doubly polished thin sections is that it gives a good opportunity to combine both fluid inclusion studies and microfossil studies. This can lead to valuable information regarding the environment in which the microfossils once lived and the physical and chemical conditions that prevailed during their lifetime. It is possible to obtain information about prevailing temperatures and fluid composition, e.g., salinity of the fluids by performing microthermometry on fluid inclusions. Normally, fluid inclusions are relatively abundant in secondary vein-filling minerals, and thus it is possible to perform such studies. Although two phase inclusions usually do not homogenize at temperatures below 60°C, which restrict the application of fluid inclusions to mostly thermophilic microorganisms. However, fluid inclusions can contain information interesting in a microbial context that does not need to be derived from microthermometry. Fluid inclusions associated with microfossils can contain trapped hydrocarbons which may give information of its origin and indicate microbial influence. Such organic compounds can be analyzed and identified with methods like Raman or some extraction technique connected to ICP-MS to distinguish a possible microbial isotopic fractionation.

### Meeting the criteria for biogenicity by using doubly polished thin sections

In trying to fulfill the criteria of microfossil recognition, the use of doubly polished thin sections can be of great support and assistance. Criteria 1, 2, 3, 5 and 6 can be more fully satisfied using this technique. Criterion 1, geologic context, can be considerably better studied and explained in detail by using all the advantages that doubly polished thin sections offer, such as fluid inclusions and better mineralogy studies. By combining fluid inclusion data with microfossil observations it is possible to determine whether a geological context is compatible with life or not. Criterion 2, indigenousness and syngenicity of the microfossil with the rock can be clarified by just using thin sections. The advantages of better visibility, better light conditions and an increased three-dimensional view of doubly polished thin sections ease the study of both the host mineral and the substrate it is attached to. Criterion 3, evidence of microbial morphology, can be much better studied due to the visibility advantages of doubly polished thin sections and the capability to view the sample from both sides. Both advantages lead to better three-dimensional visualization that simplifies morphological studies. Criterion 5, evidence of bacterial colonies or communities can be much better studied due to the greater thickness and higher level of visibility of doubly polished thin sections in comparison to ordinary thin sections. Criterion 6, occurrence of biominerals, is much easier to fulfill due to the enhanced capability of mineralogy studies that doubly polished thin sections offer. The advantages of better light conditions and higher visibility ease the study of bacterial filaments and associated mineral phases like iron-oxides or magnetite, which are well-known microbially produced mineral phases.

To meet the fourth criterion, the question of biogenicity, elemental analysis of the microfossil is required. SEM and microprobe analysis are applicable for surface analysis or to analyze a few microns below the surface. These methods are usable when analyzing ordinary thin sections or the surface of the rock sample. If, however, the sample needs to be penetrated as far as ~100 μm, which is the case with doubly polished thin sections, these methods will not work. One method that could be of use in this case is laser ablation ICP-MS. This is a method that could both penetrate the sample to a required depth and perform isotopic analysis of the microfossil. Carbon isotopic data is sometimes crucial to distinguish between a biogenic or an abiogenic origin of putative microfossils. However, since laser ablation is a destructive method, most of the microfossil will be destroyed in the analysis process.

There is, however, a way around these analytical problems and approach the question of biogenicity by returning to criterion 1 and using the full capacity of doubly polished thin sections. By combining fluid inclusion analysis with microfossil studies it is possible to place the microfossils in a geological and environmental context and find out whether the samples represent favorable conditions for microorganisms or not. It could also be used to determine whether an object is biogenic or abiogenic in origin. Abiomorphs are only known to be produced under conditions that are altogether rather rare in nature like alkaline environment, ambient temperature and from specific sources like silica, carbonate, barium ions and simple organic precursors [10]. It is possible that similar abiomorphs may form under other conditions but this has not yet been proven. By using fluid inclusions it is possible to determine the environment in which the putative microfossils existed and, thus, to conclude whether such conditions represent favorable environment for abiomorph formation or not. Thus it should be possible to rule out the possible presence of abiomorphs in some samples. This is of course not only applicable on samples of volcanic origin. Doubly polished thin sections can be prepared from all kinds of rocks including sedimentary rocks and, thus, for example, may be used as a tool for determining biogenicity of the disputed Archean microfossils [13,14]. The alleged hydrothermal setting of the Apex cherts could probably be much better studied and understood by studying fluid inclusions. The recent investigation of fluid inclusions from the ~3.5-Gyr-old Dresser Formation at the North Pole area in Pilbara craton, Western Australia is a good example of such a study. There, Ueno and co-workers [25] have found fluid inclusions that contain microbially produced methane with carbon isotopic compositions less than -56‰, which indirectly indicates the presence of methanogenic microbes.

## Conclusion

The use of doubly polished thin sections in the study of microfossils in volcanic rock leads to several advantages compared to the use of ordinary thin sections. Better visibility and light conditions, variability in thickness of the thin sections, the ability to view the sample from both sides and the possibility to combine fluid inclusion studies with microfossil studies result in great advantages in the study of morphology, internal microstructures, microfossil colonies, interactions between the microfossil and the associated substrate and finally the geological and environmental context in which the microfossil once lived. These advantages are of great importance in trying to meet the criteria for microfossil recognition. In particular the combination of fluid inclusion studies and microfossil studies can be crucial in helping distinguishing between biogenic microfossils and abiotic abiomorphs.

**Table 1:** Results of element analysis using a Philips XL 30 ESEM-FEG scanning electron microscope and a Philips EDAX (EDS) instrument. Filament 1 is the filament showed in figure 2A, B and figure 4. Filament 2 is the filament showed in figure 2C and D. Filament 3 is the filament showed in figure 2E and F. Filament 4 is the filament in 3A. Filament 5 is the filament in 3B and C. The filaments are enriched in C, compared to the volcanic glass they are attached to and similar in carbon composition to the calcite it is embedded in. One filament is enriched in C relative to the calcite. The enrichments in C indicate the biogenic origin of the microfossils. The filaments show a chemical conformity despite the fact that they occur at various depths and that they originate from three different seamounts. Slight variations between the elements occur but K and Ca are the only elements that are exchangeable.

**Elements**	**Volcanic glass**	**Calcite**	**Filament 1**	**Filament 2**	**Filament 3**	**Filament 4**	**Filament 5**
C		13.77	11.99	9.12	10.24	18.29	9.33
O	35.60	51.51	37.90	38.24	42.25	39.02	39.93
Fe	10.55		8.68	9.95	8.54	13.62	7.68
Na		1.93	9.36	6.55	1.26	1.64	1.49
Mg	0.75	1.19	3.57	3.88	6.25	3.86	4.77
Al	6.25		6.84	7.70	5.67	5.83	8.21
Si	37.59		19.70	22.20	21.53	17.74	26.41
K	4.52		1.96	2.37	1.27		2.19
Ca	4.73	31.60			2.98		
Total	100.00	100.00	100.00	100.00	100.00	100.00	100.00
